# “*The Bitter Laughter*”. When Parody Is a Moral and Affective Priming in Political Persuasion

**DOI:** 10.3389/fpsyg.2016.01144

**Published:** 2016-08-09

**Authors:** Francesca D’Errico, Isabella Poggi

**Affiliations:** ^1^Psychology Faculty, Uninettuno UniversityRome, Italy; ^2^Fil.Co.Spe. Department, Roma Tre UniversityRome, Italy

**Keywords:** parody, deep and surface parody, moral emotions, evaluation, political persuasion

## Abstract

Research on socially aware systems requires fine-grained knowledge of the mechanisms of persuasion in order to promote civic knowledge and aware political participation. Within humor studies, political parody is generally considered a simple pleasant weapon for political evaluation, currently explained by referring to the so called “just a joke effect” (Nabi et al., 2007). Indeed the funny side of parody can induce positive emotions, but it also includes a discrediting act that sometimes produces a “bitter laughter.” The present study aims to understand the role played by negative and moral emotions aroused by parody. A parody is defined as a communicative behavior (a discourse, text, body movement, song) that imitates a communicative behavior or trait displayed by some Target by reproducing it in a distorted way, with the aim of making fun of the Target. Based on a socio-cognitive approach, a distinction is made between “surface” and “deep” parody (Poggi and D’Errico, 2013), with the former simply imitating behaviors actually displayed by the Target, and the latter implying a (humorous) re-categorization of the Target. The paper studies the effect of these two different types of parody on persuasion processes. Results show that the *deep parody*, as opposed to *surface parody*, triggers more negative emotions, and in particular indignation, that in turn lead to more negative evaluations of the Target. Moreover, the moral priming of parody is influenced by the Target politician’s gender.

## Introduction. Evaluation and Emotion

Persuasion is the art of inducing people to do things by convincing them that what you propose is the right thing to do (Poggi, 2005). This process, aimed at causing a change of mind and possibly a decision making in the other, implies leading the other to conceive of new particular evaluations: “right” means that one assesses a job to choose, a product to buy, a policy to take, a candidate to vote, by making reference to some criterion of evaluation, some goal with respect to which that is the most adequate decision to make, or at least one better than another.

Affect is another process often implied in persuasion, since our final choice to do something heavily depends on the emotions we feel about the entailed object, action, situation.

These two aspects are pointed out in Aristotle’s model of persuasion, according to which the Audience is persuaded not only through *logos* (the orator’s discourse), but also through *pathos* (the audience’s emotions), and *ethos* (the orator’s personality).

The effectiveness of pathos – the appeal to emotions – is well-known ever since classical rhetoric, that solicits the orator to “*movere et delectare*,” to “move and amuse” the audience. The importance of *ethos*, the orator’s personality, accounts for why, for instance, politicians on the one side try to project the best of their image during speeches and debates, while on the other try to cast discredit over opponents: because spoiling the others’ face undermines their persuasive potential.

A particular case in which criticism is conveyed and discredit is cast over politicians is political satire, and within it, parody. One can make a parody of a song, a film, a poem, or, finally, of a person. The parody of a person (Target) is a distorted imitation of some trait or behavior of the Target aimed at amusing the audience and at making fun of that trait or behavior, or of the person *per se*. And making fun of something or someone is a way to cast discredit over it.

Therefore, political parody often has a deliberate persuasive effect: to make the Target be evaluated negatively by the Audience, the political critic (the satirical writer or comedian) makes a parody of the Target to let him/her appear less smart, altruistic, or strong than s/he tries to look, so as to weaken his/her political appeal.

This paper explores the affective and evaluative processes underlying the reception and comprehension of a political parody, and the effects of different types of parody in terms of the emotions triggered and of the evaluations elicited in the audience.

## Related Work

Within previous work on Parody, Holman and Harmon (1986) define it as an imitation intended to ridicule or criticize, that to be understood requires familiarity with the original object, and to be effective must sound faithful to the original. Rose (1979, 2011) sees parody of literary works as the comic reworking of preformed material through their partial imitation or evocation in a comic manner that marks the ambivalence of the parodist’s attitude to the object of criticism. A parody contains two texts-worlds, and the reader must understand the comic-satiric relationship between them (Condren et al., 2008; Rose, 2011; Davis, 2013). Far from being a simple imitation, it is an “approximation” to an original source where, like in sarcasm, “the subject is treated in a contradictory manner: elevated subjects are debased and low ones are elevated” (Kreuz and Roberts, 1993; Condren et al., 2008; Davis, 2013). Bakhtin (1981, p. 76) views the parodistic act as “an arena of conflict between two voices” in a hostile contrast, where the second represents a “semantic authority” with which the audience is expected to agree.

To Hulstijn and Nijholt (1996), verbal parody is a situated, intentional, conventional speech act that re-presents some object but flaunts the re-presentation to convey humorous criticism; after Poggi and D’Errico (2013), like ridiculization, it is a form of “moralistic aggression” (Bischof, 1980).

Rossen-Knill and Henry (1997) mention four pragmatic aspects of parody: (1) the intentional verbal representation of the object of parody, (2) the flaunting of the verbal representation, (3) the critical act, and (4) the comic act.

The techniques exploited by the parodist to refashion an older text or image range from caricature to substitution, addition, subtraction (Rotermund, 1963), exaggeration, condensation, contrast, and discrepancy (Davis, 2013).

In the parodistic act (Rotermund, 1963; Rose, 1979, 2011; Luttazzi, 2001; Davis, 2013), the interaction between parodist and audience is successful only if the audience acknowledges the parodist’s authority and moralistic intention; but this also requires knowledge of the target’s vices and virtues, especially when the focus are his/her body and verbal features (tics, stuttering…) that are the trigger of the comic part.

Political parody is a case of political satire, that is, a communicative act aimed at eliciting laughter in order to discredit a politician or a political party or ideology by making fun of it, often aiming in turn at political persuasion (D’Errico and Poggi, 2013). According to persuasion research, humor, when used to discredit a person, is likely to persuade via the “peripheral route” (Petty and Cacioppo, 1986), and through inhibiting counter-arguing (Nabi et al., 2007; Baumgartner et al., 2012). The crucial feature of the peripheral route to persuasion is that the stimulus is processed in a basic form, by eliciting only simple inferences, as opposed to the central route, that on the contrary involves higher motivation, more fine-grained message processing and evaluation of the source’s arguments, thus finally producing a more enduring attitude change.

Within this framework, Nabi et al. (2007), in the mass media domain, study parody and the function of humor in it in terms of its effects on attention, comprehension, credibility and judgment: by investigating the “just a joke effect,” they show that humor, while attracting attention, can promote the peripheral process of persuasion since it causes distraction from critical parts of the message. They find that humor promotes funnier messages, a more enjoyable source, and less counter-arguing: a processing chain they call “sleeper effect.”

In contrast, Baumgartner et al. (2012) reveal the “priming effect” of satire, taking the so called “Fey effect” as an example: during the Bush vs. McCain presidential campaign of 2008, the satire of Sarah Palin made by the satirist Tina Fey reduced the political evaluation of Palin in both republicans and democrats, but mostly among older electors. The variability in the effect of humor is due to the wide range of humoristic genres and cultural differences, which can be understood if we consider the description of *psychological processes* and *message features* involved in parody persuasion. As to psychological processes, the most investigated one is the “perceived funniness” on the part of the audience, that implies a process of absorption and distraction from the critical part of the parody; this causes a decrease in counter-arguing, hence higher agreement on the critical part of it (for a review see also Poggi et al., 2011). From this point of view parody can be seen as a “discounting” mechanism that causes a negative evaluation of the parodied politicians, but perceived funniness counterbalances this negative effect (Matthes and Rauchfleisch, 2013). The audience, when exposed to parody, is focused on the comic message because it provides humorous pleasure, but reduces scrutiny of the criticism borne by the message (Young, 2008; LaMarre et al., 2014).

In particular such discount effect is noticeable when viewers are young and have low “political” knowledge (Boukes et al., 2015), because they do no have enough competence to grasp indirect meanings within the parodistic message. The authors specify that actually political parody differs from “daily parody” in terms of explicit reference to daily situations and events: this is why the latter can imply less cognitive effort. As to the message features, Holbert et al. (2011) distinguishes two types of satire, a horatian and a juvenalian one, and points out how they can differently affect emotions and hence counter-argument: the horatian satire can be considered a comedy, a lighter form of satire than the juvenalian one, that can be more acid in tone (D’Errico and Poggi, 2014), more “savage and merciless” (Sander, 1971); the horatian parody is evaluated more funny by persons with low political ability, whereas high ability persons prefer juvenalian parody, and this also affects their possibility to counter-argue. Boukes et al. (2015) too compares “gentle” to “harsh” forms of parody, highlighting how lighter forms of humor are less persuasive and “critical,” because they are based on lighter cognitive effort and more humoristic pleasure. Finally, another variable that can activate the disengaged cognitive route is the *political congruity* between candidate and audience: Boukes et al. (2015) in fact noted that an initial affect toward the politician discourages the critical evaluation and then only promotes the idea of funniness of the comic message.

On the basis of the above studies it seems useful to consider different types of humor and parody and specify their different features, as Holbert et al. (2011) does.

## Parody as Moral and Affective Priming

An underestimated determinant of the effectiveness of parody in terms of political persuasion are emotional factors, and in particular the effect of negative and moral emotions (Tangney et al., 2007; Castelfranchi, 2015; D’Errico and Poggi, 2016) caused by different acts of discredit.

Emotions, in fact, can be considered as a first evaluation of parodies, and in particular the negative ones can be an alert in a “moral” sense: for example, indignation and contempt toward a discredited politician, especially when a parody heavily emphasizes potential harm and negativity, can have a role in the persuasion process. Several experiments demonstrated that affect is associated to a network of coherent memories (Bower et al., 1981) and also that negative emotions and mood can lead to more systematic information processing (Sinclair and Mark, 1995; Forgas, 2007); in the same vein, the negative and unmoral information carried by a critical parody can elicit negative emotions and so moral schemas (Ben-Nun Bloom, 2014) that can favor a more analytic processing and strict evaluation of the politician’s behavior. More specifically, Ben-Nun Bloom (2014) suggested that moral emotions like disgust increase moral conviction and a harder moral judgment, especially among political opponents.

Starting from these experimental evidences, the goal of our work is to test if parody – or particular types of it – can work as a moral and affective priming in political persuasion. In this sense we presume that political parody can be a moral priming since, bringing out a target’s flaws, it indirectly elicits moral standards and then negative moral emotions. In the remainder of this paper we illustrate an experimental study mainly investigating this issue. But before going into it, Section “Evaluation, Discredit, and Ridicule” illustrates how the notions of evaluation, discredit, satire, and parody are defined in the socio-cognitive model from which our hypothesis stems.

## Evaluation, Discredit, and Ridicule

A communicative behavior frequently exploited in the political arena by political opponents – whether politicians themselves, or political critics (journalists, satiric writers, comedians) – is one of casting discredit over each other. According to a socio-cognitive model of mind, emotions, social interaction, and communication based on the notions of goal and belief (Castelfranchi, 1995; Miceli and Castelfranchi, 2000; Poggi, 2007; Poggi et al., 2011, 2012; Poggi and D’Errico, 2013), to cast discredit over some X means to spoil the image of X before some people (D’Errico et al., 2012; D’Errico and Poggi, 2013). X’s image is the set of evaluative and non-evaluative beliefs that people have concerning X (Miceli and Castelfranchi, 2000). An evaluative belief (evaluation) is a belief about whether and how much some object, event or person has (and hence may provide someone with) the power to achieve some goal. While planning to achieve our goals we generally evaluate anything and anyone with respect to several criteria (i.e., goals), utilitarian, moral, esthetic, judging it as good/bad, useful/useless, beautiful/ugly, intelligent/stupid… We evaluate something positively when it has/gives us the power for some goal, and negative either when it lacks power (inadequacy) or when it has the power of thwarting goals (noxiousness; Miceli and Castelfranchi, 2000), e.g., “stupid” vs. “bad.”

In the political contest, discredit of politicians is often exploited by other politicians, journalists, comedians in order to political persuasion. Since we are persuaded by what a Persuader tells us (*logos*), but also by how the Persuader is (*ethos*), every politician tries to project a positive image of himself, while not to have people persuaded by his opponent, he may spoil the opponent’s image (discredit him) against three criteria (D’Errico et al., 2012): *benevolence* (trustworthiness, disinterest, honesty, morality), *competence* (expertise, knowledge, reasoning, and planning skills), and *dominance* (skill of winning in competitions, influencing others, imposing one’s will). Discredit may be cast through various “discrediting moves”: accusation, criticism, insult, and ridiculization (Poggi et al., 2012; D’Errico et al., 2014).

Ridiculization – making fun of another – is a Sender P’s communicative act that conveys a negative evaluation of a Target T before an Audience A, aimed at “moralistic aggression” toward T (Bischof, 1980; Castelfranchi, 1998; Poggi, 2011; Poggi et al., 2012), in order to sanction his a-social behavior, sometimes even with pedagogical functions. In making fun of T, P conveys a negative evaluation of T for lack of power, that contrasts with T’s pretense of superiority. Such contrast between pretense of power and actual lack of power, that is, though, not threatening for P, can elicit laughter, thus resulting in a sense of superiority. P’s and A’s laughing after T together causes three effects: (a) P and A feel superior to T, being above his inadequacy, and not threatened by it; (b) this common superiority strengthens the social bonds between P and A, through the shared emotion of laughing together, their feeling similar to each other and different from T, and a sense of alliance and complicity; (c) with image and self-image attacked, T experiences shame, humiliation, abasement, s/he feels different and rejected. All this may be P’s deliberate intention, and is the social function of ridiculization. This is why making fun of another is a peculiar discrediting move, typically exploited by political satire, that has parody as one of its weapons.

## A Socio-Cognitive Model of Parody

In terms of the notions above, a parody can be defined as a communicative act – a text or a verbal or multimodal communicative behavior (a discourse, a poem, a song, a film, a fiction) – that performs a distorted imitation of another text or multimodal behavior, with the aim of amusing and eliciting laughter about either the behavior or the one who performs it. A possible goal of such eliciting laughter and amusement can be to make fun of that person or behavior aiming to cast discredit on it. A text, a discourse, a rite, an institution, and finally a person may all be an object of parody. Often this teasing practice is exploited to achieve that abasement of power that exorcizes hate against powerful people and protects them from rebellion of those in a lower position: therefore, just like pupils make parodies of their teachers, satiric comedians make parodies of politicians, sometimes simply to have fun, sometimes to highlight their political or human flaws, and thus to lower other people’s compliance with them.

### Parody as Distorted Imitation

In the parody of a person, the Parodist P imitates a Target T by reproducing his/her traits and/or communicative or non-communicative behaviors, but in a distorted, for example an exaggerated or misleading way, that highlights the Target’s flaws; to do so the parodist must single out the most characterizing features of T’s physical traits or behaviors, and imitate them while exaggerating or anyway changing them in such a way as to make them appear ridicule (Poggi and D’Errico, 2013).

The imitation performed in a parody is distorted because the goal of the Parodist is to highlight the Target’s flaw: therefore the Target’s traits are exaggerated and made grotesque.

Actually, since the imitation is distorted, the need to make the Target recognizable is even stronger than in bare imitation. So the parodist must effectively convey two distinct kinds of information, one concerning the very identity of the Target, and one regarding the features subject to criticism: while the latter must be distorted, the former must be imitated the most faithful way. In both he can do so with the help of allusion, that is, letting the Audience infer the information he refers to, but without mentioning it explicitly. Generally the Target is made recognizable through a suit he typically wears, a make up that makes the Parodist’s face and hair more similar to the Target’s, or the imitation of his voice and regional accent. As to the flaw, since often it has been evidenced by a particular event or episode, it is evoked through allusion to that episode. For example, in a parody pointing at the organizational unsuitability of Rome Mayor Gianni Alemanno during an unprecedented snow, to remind that the event occurred in Rome the Parodist Max Paiella, imitating Alemanno, is presented on the background of Coliseum.

### Surface and Deep Parody

Yet, there are two ways to distort the imitation, differing for the extent to which the Parodist’s traits or behaviors are far from the Target’s actual ones. These two different levels of distortion result in two different types of parody, that as distinguished in previous works (Poggi and D’Errico, 2013) we call “surface” and “deep” parody.

An example to clarify the difference is in the parodies of an Italian right-wing politician, Renato Brunetta, who is very short and very arrogant and aggressive against leftists. In one of them the (left-wing) comedian Maurizio Crozza simply represents him by standing on his knees. This way he simply exaggerates the trait of having short legs. Other behaviors typical of Brunetta, that give the idea of his aggressive and arrogant communication and can be easily distorted by simple exaggeration, are his habit of urging the interlocutor by repeating the same word or statement two or three times, and the gesture of approaching his index fingers while arguing. To perform a “surface” parody, that is, a simple distortion by exaggeration of these behaviors, it is sufficient for Crozza to repeat the same word many times, or do the same gesture of index fingers approaching more often than usual, even at points of the discourse in which Brunetta would never make that gesture.

Yet besides doing so, Crozza also makes “deep” parodies of Brunetta: for example when he represents him with a gun and a helmet while groveling on the ground like a guerrillero. In this case, to render Brunetta’s aggressiveness he performs a re-categorization of him: he finds a category of persons that is typically characterized by this trait, guerrilleros, and shifts Brunetta from the category of politician to this new category.

One more “deep” parody by another comedian, Max Paiella, of Fabrizio Cicchitto: this politician, formerly a most devoted member of the left-wing Socialist Party and of his leader Bettino Craxi, later became a Member of Parliament in a right-wing party, Forza Italia, and proved totally devoted and submissive to its leader Silvio Berlusconi. Paiella (in 2011, with Berlusconi being the Prime Minister) represents Cicchitto on the background of the luxurious Hall of the Italian Parliament, wearing, below his jacket, a long white pinny, and while listing the laws to be approved as if they were the items of a restaurant menu.

Let us track the cognitive processes leading to this parodistic representation of Cicchitto. First the political Parodist singles out the Target’s characterizing feature to make fun of (the political flaw): his being a very submissive politician. Then he finds a category of persons different from one the Target belongs (politicians) and prototypically characterized by that feature (the category of waiters); so he represents a Parliament member as a waiter. Such “deep” parody consists in a re-categorization of the Target, from his own category to another that has the Target’s flaw as its most characterizing feature.

Therefore, the ideation of a deep parody as opposed to a surface one implies a more complex cognitive process both on the production and the comprehension side. The Addressee of a deep parody, to fully understand its implications, starting from the re-categorization of the target (waiter) must retrieve its characterizing feature (submissive) and apply it to the actual category of the Target (Parliament members), and finally to the Target himself (Cicchitto is submissive).

Once caught the analogy between the two categories, the Addressee should map each feature of the new category onto the Target’s, seeing the waiter as Cicchitto, the restaurant as the Parliament, the meals of the menu as the laws to be approved, and the Chief of the Restaurant, who determines the meals to be cooked, as Berlusconi, who decides the laws to feed to the people. Only once reconstructed all this information can the Addressee fully understand the political criticism borne by the parody.

## Parody as Affective/Moral Priming in Political Persuasion. An Experimental Study

The goal of our work is to investigate the effects of surface and deep parodies on the Addressee, in terms of the emotions elicited and of the resulting evaluation of the Target.

### A Study on How Parodies Affect Emotions and Target Evaluations

We conducted an experimental study on different types of parody, focusing on three research questions:

(1) Our first aim was to test the psychological reality of the distinction between surface and deep parody, and to demonstrate that both imply different processes from bare imitation.
(2) Our second hypothesis is that surface and deep parody differ for the kinds of emotions they elicit, and/or for their relative intensity.
(3) The third hypothesis is that surface and deep parody have different effects on the evaluation of the Target more specifically, we hypothesized.
(4) That negative and moral emotions cause a more negative evaluation of the Target, compared to positive emotions or non-moral emotions.
(5) That deep parody, as opposed to surface parody, can promote more critical thoughts toward politicians and concerning politics in general.

### Materials and Methods

#### Experimental Design

To test these hypotheses, we designed a monofactorial between subjects experiment with one independent variable with three levels being three different representations of the same Target politician: a bare imitation, a surface parody, and a deep parody. The dependent variables were the emotions elicited in the Addressee, and the induced evaluation of the Target, possibly including negative and critical thoughts.

Hundred and eighty three participants were submitted a semi-structured on line questionnaire after viewing a video according to three different conditions corresponding to the independent variable “type of representation” (simple imitation – as a control variable – surface, and deep parody).

The sample was balanced and composed by 53% women, the majority having a high school degree (54%) or a University degree (26%), age of 37 (*SD* = 15,9), and political orientation mainly oriented toward left party (49%), compared to 20% oriented toward right party, 15% oriented to “five stars movement” and 14% to center-party.

#### Stimulus Material

Three video-stimuli were selected among a wider range of videos of Italian imitators and parodists downloaded from youtube; they were analyzed by two independent judges as to their multimodal communication (Poggi and D’Errico, 2013), and classified as imitation, surface, or deep parody, based on the following conditions:

1. For simple imitation: (1) faithful reproduction of bodily and verbal traits and behaviors typical of a real politician; (2) comic act;
2. For surface parody: (1) imitation of some bodily and verbal traits and behaviors typical of a real politician; (2) distortion of the imitation; (3) comic act; (4) critical act; (5) possible reference to a specific event triggering the critical act;
3. For deep parody: (1) imitation of some bodily and verbal traits and behaviors typical of a real politician; (2) re-categorization of the politician; (3) imitation of a scenery and/or body and verbal traits and behaviors characterizing the new category; (4) comic act; (5) critical act.

The videos selected by taking into account the conditions above were:

1. Condition 1, simple imitation: an almost unknown imitator (actually, a right-wing member of Parliament) faithfully imitating the right-wing Minister of Economy, Giulio Tremonti. His most faithfully represented features were gray hair and his accent and pronunciation, characterized by his lisp “r”;
2. Condition 2, surface parody: Tremonti imitated by the left-wing comedian Corrado Guzzanti while computing, in a very hectic way, on an electric calculator (an allusion to his being the Minister of Economy) and repeatedly uttering a curse that, including an “r”, gave Guzzanti a chance to funnily exaggerate Tremonti’s lisp “r”;
3. Condition 3, deep parody: Guzzanti, representing Tremonti, dressed in a costume of the 18th century, then re-categorized as a French King (perhaps Louis the XVI), bored to hear the people’s voices out of his sumptuous palace, and uttering sentences like Marie Antoinette’s at the time of the French Revolution. Tremonti was so re-interpreted as an autocratic king totally careless about the people’s economic problems.

All participants, assigned each to one of the three conditions, after viewing the corresponding video, were asked to fill in a questionnaire of 34 open and multiple choice questions, investigating the emotions and evaluations elicited by the video (along with cognitive aspects like comprehension of its meaning and recognition of the character, that is the object of another work, D’Errico and Poggi, 2016). At the beginning of the questionnaire, a distinction was explicitly made between the fictional character represented by the imitator or parodist (let us call it C, for example the funny Tremonti pressing on the keys of the calculator) and the Real Person represented (RP: the real Tremonti, also called the Parodied Politician). Quantitative questions asked to rate the investigated aspect on a 5 point Likert scale. Questions on emotional aspects investigated the extent to which emotions were elicited by the video (joy, bitterness, sadness, interest, amusement, boredom, pleasure, enthusiasm, displeasure, indignation) or felt toward the Character (pity, tenderness, sense of pathetic, contempt, sympathy, antipathy, indignation, admiration), and asked what moments in the video, if any, were comic. Other questions asked to evaluate the video (well done, ugly, expressive, hard, strong, amusing, exciting, exceptional, delicate, elegant, attractive, interesting, moving, pleasant), the Character and the Real Person (negative, indifferent, amusing, proactive, competent, dangerous, strong, credible, enthusiasm inducing, stupid, convincing, false, charismatic, astute).

A final question inquired the Participant’s political orientation.

### Measures

#### Manipulation Check

The manipulation of the type of parody was checked by a multiple choice question “How would you classify this video?” with possible answers (1) an imitation, (2) a simple parody close to reality, (3) a complex parody far from reality. A *chi square test* evidences a significant effect [χ(3) = 39,54; *p* > 0.000] of correct answers: the video in condition 1 was classified as imitation by 64% of participants (vs. as surface parody by 36%, as deep parody by 10%); in condition 2, 42% classified it as a “simple” parody (32% as deep parody and 24% as imitation); in condition 3,58% classified it as a complex parody far from reality (35% as surface and 7% as imitation).

Answers to the *scales of emotions* were analyzed by factorial analysis to extract positive and negative emotions. The analysis extracts two factors (positive and negative) that explain 46% of variance; reliability alpha is equal to 0.067. In the positive scale we sum joy, fun, enthusiasm and pleasure (*M* = 2,03, *SD* = 0,96; α = 0.074) while in negative, along with sadness, we sum the negative moral emotions of bitterness (Poggi and D’Errico, 2010b), indignation and contempt (*M* = 2,59, *SD* = 1,14; α = 0.070).

The *evaluation of Character* (the represented Tremonti) and of *Real Person* (the real Tremonti, i.e., the parodied politician) was rated by 14 items that cover the persuasive dimensions of competence, benevolence, dominance (Poggi and D’Errico, 2010a). A factor analysis extracted three factors: one of competence (competent, active, credible, convincing, stupid reversed), one of negative benevolence (dangerous, negative, false, and astute) and the last one a factor of induced funniness (amusing, exciting, indifferent reversed); in the case of the character, the first factor explains the 25% of variance, 17% the second one, and the third 9%. As to evaluation of the real politician, the variance explained is for the first factor 33%, for the second (negative as to benevolence) 18% and the third 8%. For the purposes of the study we will analyze mostly the second dimension so the four items were analyzed in order to have a unique mean of “negative as to benevolence of the character” (*M* = 2,65, *SD* = 0,94; reliability analysis: α = 0.069) and “negative evaluation of the real politician” (*M* = 2,73, *SD* = 1,02; α = 0.074).

#### Negative/Critical Thoughts

The dependent variable of *intensity and target of critical thoughts* was assessed respectively by measuring the answers to the question “how much negative/positive were your thoughts after the video” and by coding open answers to the question “What are your reflections after seeing this video?” 135 out of 183 answers were coded in four categories with an increasing level of generality: (a) *no reflection* (when participants declare that they have no particular thoughts); (b) *distrust or negative thoughts on the politician* (i.e., “he is a negative person,” “he is not trustworthy”); (c) *negative thoughts or distrust concerning the socio-cultural situation* (i.e., “I’m thinking of the negative situation of our country,” “if they spent our money for their privileges our negative situation will never change”; (d) *reflections concerning politics in general* (“it reflects the real political situation, they are all lazy/slackers,” “Im thinking of politicians who defend only their own interests and not those of laypeople”).

### Hypotheses

Taking up Holbert et al.’s, (2011) conclusion concerning the different effects of juvenalian and horatian satire, our hypothesis is that parody can be an affective priming not only in a positive sense (Nabi et al., 2007) but also in a negative sense, depending on its features, namely its being a surface vs. deep parody (Poggi and D’Errico, 2013).

We first of all hypothesize that parody, different from a simple imitation, can induce a mixed mood, composed by positive emotions for the comic act, but also negative and more typically moral emotions, and more so especially when the negative features of a politician are re-categorized in terms of another extreme character with exaggerated features (i.e., a dictator, a fighter), finally characterizing him as belonging to a non-politician category, as is the case in deep parody (Poggi and D’Errico, 2013).

Our hypothesis further holds that the positive vs. negative affective priming, in its turn, can influence (1) the evaluation of the Character, (2) the evaluation of the Real Person (the parodied politician), and (3) the political judgment and associated critical thinking; in other words people tend to create congruency between their emotional state and their evaluation of other people (Forgas, 2006).

We finally predict that the affective priming, coherently with the “congruity hypothesis,” will mainly affect people with a political orientation opposite to one of the parodied politician. In our case our prediction is that, facing the parody of a right-wing politician, left-wing participants will be more affected by negative emotions and similarly more negative will be the evaluation both of the parody Character and of the Real politician.

### Results

All the presented results are significant; all other results will be not taken into account in the following description. An ANOVA analysis of Type of Parody, illustrated below in **Table 1**, shows significant differences between conditions: the positive emotions joy, fun, pleasure and enthusiasm are higher mostly in surface parody compared to imitation and deep parody, while negative ones like bitterness, contempt, indignation and sadness are higher in the deep parody condition. Surprisingly, negative emotions in general are basically higher than the positive ones (2,71 vs. 2,01; *p* < 0.05).

**Table 1:** Descriptive statistics of emotions (mean and SD).

	Imitation (*n* = 60)	Surface (*n* = 65)	Deep (*n* = 57)	Total	*p*
Joy	1,28 (0.6)	1,94 (1,2)	1,79 (1,1)	1,68	<0.002
Fun	1,93 (1)	3,09 (1.4)	3,01 (1.1)	2,69	<0.000
Pleasure	1,63 (0.8)	2,22 (1.2)	1,74 (1)	2,01	<0.05
Enthusiasm	1,38 (0.64)	1,95 (1.2)	1,89 (0.9)	1,75	<0.003
Bitterness	3,01 (1.3)	2,66 (1.3)	3,14 (1.2)	2,95	<0.05
Contempt	2,34 (1)	2,38 (1.1)	2,98 (1)	2,55	<0.05
Indignation	2,63 (1.4)	2,46 (1.5)	3,07 (1,3)	2,95	<0.05
Sadness	2,45 (1.3)	1,97 (1)	2,56 (1.3)	2,45	<0.05

In general also for what concerns the evaluation of the Character (Tremonti as represented by the parodist Guzzanti) an ANOVA shows that in the surface parody condition, coherently with the positive emotions previously described, participants evaluate the Character more positively (amusing, exciting, and also competent) than in imitation and deep parody. This result seems coherent with the so called “just a joke effect” (Nabi et al., 2007) in that the funnier the video is perceived, the more positive the Character evaluation. Furthermore, the means of the negative items, *negative, dangerous* and *astute* tend to be higher than the positive ones, and summing them we can see a good difference: 2,7 vs. 2,2 (*p* < 0.05 at *t*-test paired); only the “*stupid*” item is higher in the surface condition (coherently with the dimension of discredit evoked by the surface parody). Thus, parody remains a funny genre, but nonetheless elicits negative evaluations of the character.

As to the *evaluation of the Real Person* (the Parodied politician), the ANOVA shows that the condition affects two items only: *negative* and *astute* (**Table 2**), this last dimension evoked by the deep parody condition, where Tremonti/King Louis is discredited for his lack of benevolence. However, in the deep parody condition the parodied politician is evaluated as more negative and as more astute than in the surface and imitation condition (*negative* 3,29 vs. 2,95 in surface and 2,37 in imitation; *astute* 3,21 vs. 2,74 in surface and 2,53 in imitation condition; *p* < 0.05 and *p* < 0.05).

**Table 2:** Descriptive statistics of Character evaluation (mean and SD).

	Imitation (*n* = 60)	Surface (*n* = 65)	Deep (*n* = 57)	Total	*p*
Amusing	2,15 (0.9)	3,23 (1,2)	3.02 (1.2)	2,82	0.000
Exciting	1,37 (0.6)	1,66 (0.8)	1,95 (0.9)	1,65	0.02
Competent	2,12 (0.8)	2,34 (0.9)	1,91 (1)	2,13	0.01
Negative	2,7 (1.2)	3,22 (1.2)	3,49 (1.2)	3,13	0.003
Dangerous	1,93 (1.1)	2,52 (1.2)	2,63 (1.3)	2,36	0.01
Astute	2,48 (1)	2,52 (1,1)	3,12 (1,2)	2,63	0.003
Stupid	2,27 (1.1)	2,95 (0.9)	2,89 (0.8)	2,77	0.002

#### Parody, Emotions Elicited, Evaluation of the Character and of the Real Person: a Mediational Analysis

To outline the relation between video, emotions and evaluation of the Character and of the Real Person, we performed three significant mediational analyses. To test the mediational hypothesis, a series of regression analyses were performed following the procedures outlined by Baron and Kenny (1986).

This procedure consists of a three-step series of regression analyses. In the first regression, the independent variable is associated with the dependent variable; in the second regression, the independent variable has to be associated with the hypothesized mediator. The third phase consists of a regression where the effects of both independent variable and mediator on the dependent variable are tested. Mediation is demonstrated when the addition of the mediator variable into the third regression equation substantially decreases or eliminates the previously significant relation between the independent variable and the dependent variable. The relations involved in the mediations are also tested by means of Sobel Test and they are both significant (1) T: 2,15; *p* < 0,03; (2) T: 2,57, *p* < 0.010.

**FIGURE 1:**
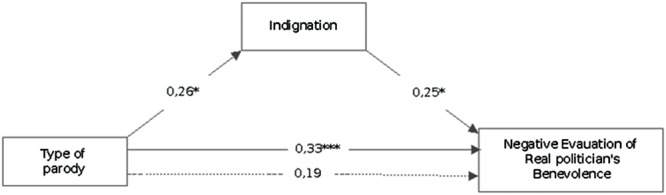
**Mediational regression analyses of type of video and indignation on negative evaluation of character’s benevolence.** The type of Video variable was processed as a progressive number 1 = imitation, 2 = surface, 3 = deep associated to the progressive “severity of parody,” so the positive relation must be read as follows: when “severity of parody” increases indignation increases too ^∗^*p* < 0.05; ^∗∗∗^*p* < 0.001. Solid lines between variables denote direct paths between two variables. Dotted lines denote paths when moral and negative emotions are included as mediator. Values denote standardized Beta weights.

**Figure 1** shows how the experimental condition (1: imitation, 2: surface, 3: deep) is significantly related to the moral emotion of indignation, the stricter the parody is, the more indignation participants feel (β: 0.026). Indignation correlates with a negative evaluation of the real politician’s benevolence (β: 0.025), and their mediation also lowers the direct relation between type of video and negative evaluation of the politician’s benevolence (p:n.s.), being a good moderator. So, the stricter the parody, the more negative moral emotions people tend to feel, and the more negative the evaluation of the parodied politician.

**FIGURE 2:**
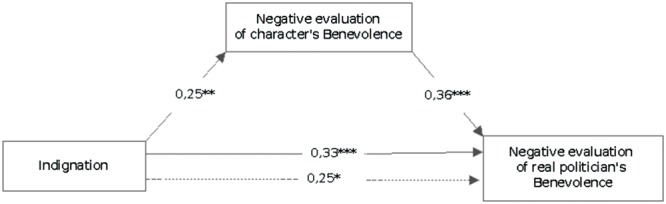
**Mediational regression analyses of indignation and negative evaluation of character’s benevolence on negative evaluation of real politician’s benevolence.** ^∗^*p* < 0.05; ^∗∗^*p* < 0.01; ^∗∗∗^*p* < 0.001. Solid lines between variables denote direct paths between two variables. Dotted lines denote paths when negative evaluation is included as mediator. Values denote standardized Beta weights.

The second mediation shows how indignation is more strongly associated to the negative evaluation of the real politician’s benevolence when they are mediated by negative evaluation of the Character’s benevolence (β: 0.033). This mediation highlights that indignation elicited by deep parody also affects the represented politician, and this evaluation can mediate the overall evaluation of the real politician: in a sense, when the indignation is high also the pleasure and funniness of the represented Character decrease (**Figure 2**).

#### Critical Thoughts and the Target of Distrust

**FIGURE 3:**
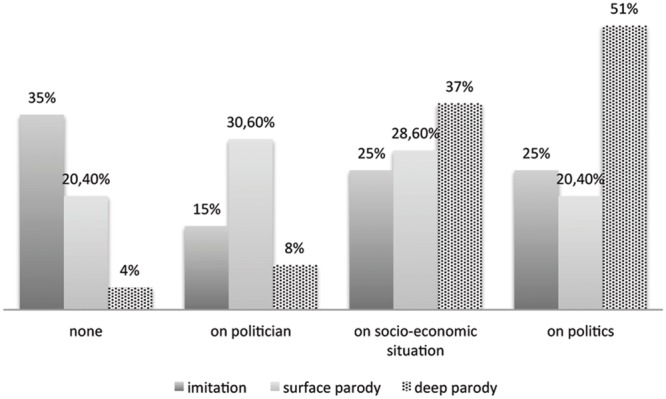
**Target of distrust^∗^conditions**.

In order to test the effect of the video on critical thinking, at the end of the questionnaire we asked “how much negative/positive were your thoughts after the video.” The differences in answers were significant to an ANOVA [*F*(2,181) = 3,696; *p* < 0.025], in that participants evaluate their thoughts more negatively in the deep parody condition than in the other two conditions (4,11 vs. 3,74 in surface parody and 3,5 in imitation) (**Figure 3**). No significant difference on positive thoughts between conditions, but an interesting result is that when comparing positivity and negativity of the participant’s thoughts we can say that, surprisingly, positive thoughts means are significantly lower than the negative ones (1,57 vs. 3,77; *p* < 0.05 at *t*-test paired).

While usually in previous experiments (Nabi et al., 2007; LaMarre et al., 2014) the researchers collected a dependent variable called “counter-arguing,” the agreement on the critical part of the parody’s message, in our study we wonder if parody affects thought not directly connected to the specific message of the parody, but more in general the amount of negative/critical thoughts toward the politician, or the overall socio-cultural situation or more in general politics. This might be read in terms of distrust and cynicism (Cappella and Jamieson, 1997; Castelfranchi, 2013). The answers to the question concerning negative/critical thinking, “What are your reflections after seeing this video?,” were coded into four categories (no reflection; distrust on the politician; distrust concerning the socio-cultural situation; distrust concerning politics in general); on the total of the codified answers across conditions the highest percentages are on the socio-economic situation (33%) and on politics in general (34%), demonstrating a general level of distrust, and political cynicism (just 15% have no particular thoughts and 18% have ones on that particular politician).

Considering the manipulation, a chi square test reports a significant effect for type of video [χ(4) = 27,51; *p* > 0.00]: in the deep parody condition critical thoughts show a higher level of generality than in other conditions. *Deep parody* promotes mainly thoughts strictly linked to distrust in politics (51%) and on the socio-economic situation (37%), but to a lesser extent concerning the parodied politician (8%) or no thought in particular (4%). On the contrary in the *surface parody* condition thoughts linked to the character (30,60%) are higher than in the other cases; in the imitation condition participants have no critical thoughts (35%).

The negative evaluation of the real politician’s benevolence significantly affects the negative thoughts at the end of the video and the target of distrust as demonstrated by a linear regression (**Table 3**): when the politician is evaluated more *dangerous, astute, negative* and *false* by participants, the level of negative reflections and general distrust tend to increase (1 no distrust – 4: distrust toward politics).

**Table 3:** Regression predicting negative thoughts and target of distrust.

	Evaluation of Real politician’s Negative benevolence		
D.V	*B*	*SE*	*p*
Negative thoughts	0.28	0.25	0.01
Target of distrust	0.46	0.33	0.04

*The Table illustrates: unstandardized coefficient (b), coefficient with standard errors (SE), and probabilities (p).*

*The Variable negative thoughts is on a Likert scale (1–5) while Target of distrust was processed as a progressive number 1 = no distrust, 2 = distrust toward politician, 3 = distrust toward socio-economic situation, 4 = distrust toward politics, so the positive relation must be read as follows: the negative evaluation of the real politician’s benevolence also increases “generality of distrust.”*

#### Political Orientation

As to political orientation, taking into account that in this study the Target politician was the right-wing Minister Tremonti, we analyzed the potential main and interaction effects on dependent variables and only two main effects resulted significant. An ANOVA shows a main effect on bitterness due to parody [*F*(3,143) = 3,052; *p* < 0.05, η^2^ = 0.45]: leftists feel more bitterness (3,16) than participants siding with the center (2,85), the right (2,8) and five stars movement (2,6). The case of right-wing participants can be explained by the “congruity effect” (Boukes et al., 2015): the more congruent the political orientation the fewer negative emotions are elicited by parody. The case of five stars movement, a party extremely opposed to the parodied politician (Tremonti) can be explained by their cynicism toward traditional political parties (but a next study will have to further investigate this). The negative evaluation of politicians too presents a significant main effect for political orientation [*F*(3,143) = 2,858; *p* < 0.035, η^2^ = 0.48]: membership in the same party as the parodied politician’s determines less negative evaluation. So leftists (3,08), center-wing (3) and five stars movement participants (2,88) present a more negative evaluation of real Tremonti than the right-wing ones (2,4) (**Table 4**).

**Table 4:** Descriptive statistics of bitterness and negative evaluation of the real politician’s benevolence (mean and SD).

	Left (*n* = 73)	Center (*n* = 20)	Right (*n* = 30)	Five stars (*n* = 25)
Bitterness	3,16 (1.2)	2,85 (1.3)	2,8 (1.2)	2,6 (1.2)
Negative evaluation of real politician’s benevolence	3,08 (1.3)	3 (1.4)	2,4 (1.3)	2,88 (1.3)

**FIGURE 4:**
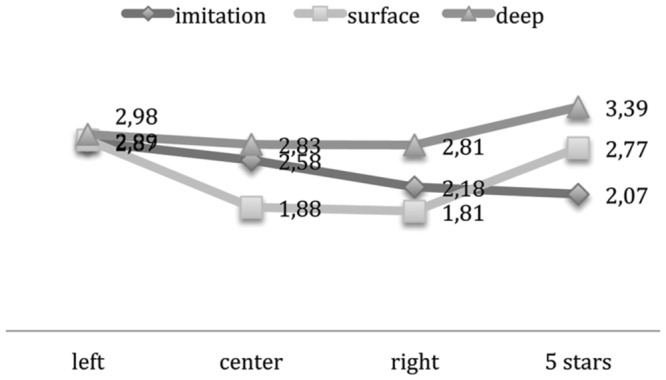
**Political orientation^∗^conditions on politician’s negative evaluation**.

These two main effects are in line with possible expectations, but an interesting result is the interaction effect between *political orientation* and *type of representation* [*F*(3,143) = 2,328; *p* < 0.025 η^2^ = 0.93] on the evaluation of the politician, that shows how deep parody determines an increase in negative evaluation particularly on the part of political congruent participants (rightists: in surface condition 1,25 vs. deep parody 2,83) different from leftists that are quite constant across conditions (**Figure 4**). We can deduct that right voters (congruent with the parodied politician) are more affected by a deep parody than left, center or five star movement voters; in this case they evaluate the politician as more dangerous, but this evaluation does not impact on their level of trust.

## Study Two. What’s the Role of Gender in the Effects of Parody?

To go more in depth into the persuasive effects of parody, we checked if results concerning felt emotions and evaluation of the character and of the real politician change when either the Parodist or the Target politician is female.

In this case, is parody still an affective and moral priming or not?

We conducted an experimental study on different types of representation, comparing the effect when the politician is a male vs. a female (performed by a same gender parodist).

Considering that the roles of power, in politicians, are mainly masculine (Lorenzi-Cioldi, 1988; Burr, 2002; Paxton and Kunovich, 2003), particularly in the Italian context (Gelli, 2009), we expect that parody as an aggressive act, will affect male politicians more than female ones: first because politics is not a female stereotypical field, so their role is seen as less relevant, and secondly because females are under-represented among politicians, so an attack on them, throught a parodistic act, can be seen just as a risible comic act:

We hypothesize that parody as an act of discredit will be less effective in female than in male condition and in particular

(1) Emotions will be more positively oriented in female parody than in male one;
(2) Evaluation of the character and of the real politician will be more positive in female than in male condition.

### Material and Methods

To test these hypotheses, we designed a bifactorial between subject experiment with two independent variables, type of representation and the politician’s (and Parodist’s) gender: the first with three leves, bare imitation, surface and deep parody, the second with two levels, male vs. female politician (and Parodist). The dependent variables and measures were, like in the first study, emotions elicited in the addressee, evaluation of the character, and evaluation of the real politician.

Two hundred and thirty one participants filled in a half-structured on line questionnaire after viewing a video according to the six conditions corresponding to the independent variables. The sample was composed by 63% women, the majority with a high school degree 64%, age of 33,4 (*SD* = 15,7) and mostly oriented toward left (37,2%; 11,7% toward center-party, 17,3% toward right party and 16,8% toward five stars movement; the remaining participants declared “no political orientation”).

### Stimulus Material

The three stimuli in male condition were the same of the first study; while in female condition we selected a right-wing female politician, Giorgia Meloni as Target of three videos, analyzed by two independent judges and coded as (1) pure imitation, (2) surface parody, and (3) deep parody.

The conditions were the following:

1. Condition 1, simple imitation: the left-wing comedian Sabina Guzzanti imitates Giorgia Meloni, right-wing candidate to Mayor of Rome, who is pregnant and whose delivery will exactly coincide with the end of the political campaign and her possible election to Mayor. Guzzanti represents Meloni while looking for a babysitter, asking for a babysitting 24 h a day, and complaining that a Rumanian babysitter (toward whom she is has racist biases) would charge her 150 euros a day for that.
2. Condition 2, surface parody: Comedian Paola Minaccioni makes fun of hectic Giorgia Meloni’s habit of showing as a super-woman: she represents her while throwing a weigh of 2000 kilos, viewing things on the back of her head, cooking a chicken in 7 min…
3. Condition 3, deep parody: Sabina Guzzanti acts Meloni, on the background of a helicopter, dressed in a camouflage like a soldier, and while complaining about an invasion by Moroccans, for instance Balthasar with his camel, who occupy even the crib.

### Results

**FIGURE 5:**
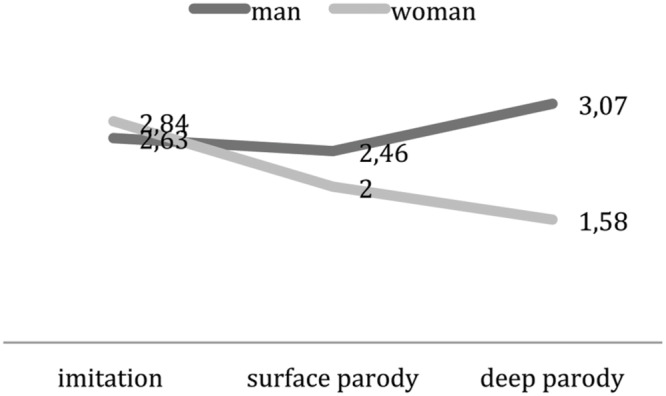
**Type of parody^∗^Parodied politician’s gender on Indignation**.

All the presented results are significant; others results will be not taken into account in the following description, except for some cases close to significance. Results concerning emotions point out that in male condition participants feel more bitterness, indignation, interest and less boredom (*p* < 0.05) than in female condition. The male parody activates participants (less boredom and more interest) and, congruent with the starting hypotheses, it causes more negative moral emotions like indignation, because politics is represented mostly as a masculine domain (Lorenzi-Cioldi, 1988) and thus it carries more expectations on male politicians (see **Table 5**). Moreover, an ANOVA points out an *interaction effect* between type of representation and gender on the moral emotion of indignation [*F*(2,230) = 4,121; *p* < 0.015, η^2^ = 0.72]: while in male condition, the stronger the parody the more indignation participants feel, in female more indignation occurs in imitation and in surface parody. This might be due to the fact that not so much aggressiveness is necessary in the parody, given that female politicians are devoid of decisional power (**Figure 5**). Among less significant results are the positive emotions, that are slightly lower than the negative ones, and higher in female condition: like in the case of joy (*p* = 0.14).

**Table 5:** Descriptive statistics of emotions^∗^ Parodied politician’s gender (mean and SD).

	Man (*n* = 182)	Woman (*n* = 48)	Total	*p*
Bitterness	2,95 (1.3)	2,17 (1,2)	2,56	<0.000
Indignation	2,71 (1.3)	2,23 (1.4)	2,47	<0.05
Interest	2,5 (0.9)	2,02 (1.1)	2,26	<0.05
Boredom	2,13 (1.2)	2,54 (1.3)	2,33	<0.05

**FIGURE 6:**
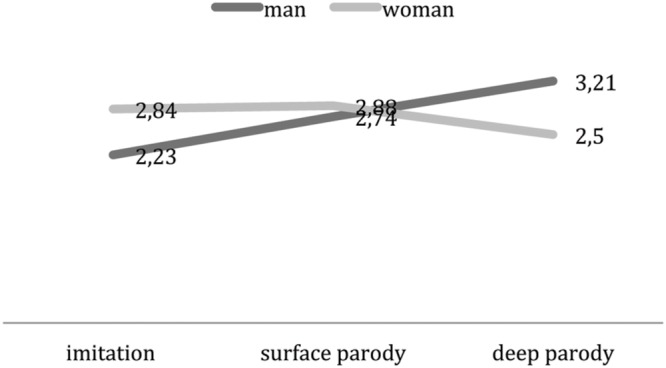
**Type of representation^∗^Parodied politician’s gender on Astute**.

The gender trend in parody is also confirmed by results on the evaluation of the character and of the real politician, because negative – and dangerous – features are attributed, regardless of the type of representation, more to the male character than to the female one (**Table 6**). Evaluation of the real politician too, after viewing the parody, is more negative and less amusing in male condition than in female condition. Moreover, also in this case another result close to significance is the positive evaluation of the character, seen as more exciting in female condition (*p* = 0.11), even if this is lower than for negative items (1,65 in male condition vs. 2.35 in female one). In a certain sense, parody as a moral priming seems more effective on male politicians, while in women it remains just a joke. In particular in male condition, deep parody affects the evaluation of the real politician as more astute (3,21) than in imitation and surface parody (2,23 and 2,88), while the female is evaluated astute in the average across conditions. This interaction effect is significant at an ANOVA analysis [*F*(2,230) = 3,564; *p* < 0.05, η^2^ = 0.61] and it demonstrates that enhancing discredit toward a politician by means of parody, worsens the evaluation of male politicians and to a lesser extent that of female ones (**Figure 6**).

**Table 6:** Descriptive statistics of character and real politician evaluation^∗^ Parodied politician’s gender (mean and SD).

	Man (*n* = 182)	Woman (*n* = 48)	Total	*p*
**Character evaluation**				
Negative	3,13 (1.3)	2,75 (1,2)	2,94	<0.05
Dangerous	2,36 (1.3)	1,96 (1.2)	2,16	<0.05
**Real politician evaluation**				
Amusing	1,73 (0.9)	2,08 (1.1)	1,9	<0.05
Dangerous	2,47 (1.2)	1,85 (1.3)	2,16	<0.001

## Discussion

The goal of this study was twofold: first to investigate the persuasive effects determined by two types of political parody, surface and deep, differing for their multimodal – bodily and verbal – arrangement (Poggi and D’Errico, 2013) but, before that, by their different cognitive structure. Surface and deep parody respectively result from different levels of distortion stemming of a different categorization of the behavior or personality of the parodied politician. In surface parody the distortion is relatively light since it lets the Target be clearly recognizable as a member of his source category; in deep parody instead, distortion gives rise to a re-categorization (Rosch, 1973), hence to a different person, for instance one disguised as an absolutist king or a submissive servant.

In the second place, while the great part of literature mainly points at the role of positive emotions in the discounting process of parody, our goal was to investigate the role of negative and moral emotions in the evaluation of the politician and in the consequent persuasive effect. Results evidence how parody in general elicits more negative than positive emotions, and how the two types of parody elicit them in different ways. While surface parody mainly elicits positive emotions and presumably a “just joke effect” (Nabi et al., 2007) thanks to its focusing on the comic effects of parody, deep parody mainly elicits negative emotions like sadness and bitterness, and moral emotions like indignation and contempt. Surface and deep parody therefore exert a different affective priming that also reverberates on the evaluation of the Character: while surface parody makes him appear more amusing and exciting, deep parody (that in our case mainly discredits the Target’s benevolence) makes the character less amusing but more competent, dangerous, negative and astute. Moreover, results clearly evidence how participants, after viewing the deep parody, evaluate the Character as more negative and astute than the Real Politician.

The relationship among types of parody, negative emotions and Character evaluation has then been made explicit by a mediational model (Baron and Kenny, 1986): deep parody elicits negative moral emotions, in particular indignation, that turn into a negative evaluation of the Character (**Figure 1**), further significantly related with a negative evaluation of the Real Politician. Such relation is stronger depending on political congruity (Boukes et al., 2015): in our case in fact negative emotions are experienced more by leftist participants, of the side opposite to the parodied politician.

So, it is interesting to highlight how a parody, when deep, can be a moral priming, due to its eliciting moral emotions such as outrage, and exacerbates evaluative processes (Ben-Nun Bloom, 2014; Ruch and Heintz, 2016) toward politicians and also politics, increasing the tendency to distrust.

One more issue we explored are the thoughts associated to the affective state induced by the two types of parody: when asked about their reflections after the video, participants in the surface condition mainly expressed negative and critical thoughts, hence distrust toward the politician and the socio-economic condition, while in the deep condition, negative thoughts concerning the socio-economic situation, but mainly toward politics in general increase.

Surface parody, keeping its representation within the source category, looks more informative and closer to the real politician, hence inducing critical and distrusting reflections on the Character, Minister Tremonti represented while computing on his calculator. Instead, Tremonti represented in the category of an absolutist King despising the people out of his door increases indignation and contempt, hence distrust in the whole political class. Thus evoking a higher level of cynicism.

A particular mention is to be done concerning *political orientation*, from which no significant results emerge to confirm a strong difference between rightist and leftist groups (Bonferroni test). This seems due to the peculiarities of the parodied character, who belongs to the right, but is not presently part of the leading group of the party; moreover, Tremonti was considered as a cold “technician,” being an University professor of economy before becoming a right minister, not so beloved by right voters. In right-wing participants, the deep parody has the effect of increasing the negative evaluation of the politician, seen as astute, whereas the leftists maintain a generally negative assessment of the conditions. Future work may choose a parody of a politician strongly supported from his own party, which makes us expect a greater polarization.

In the second study we also tested if the politician’s gender morally affects participants’ emotions and evaluations across parody conditions; results demonstrate that parody determines the perception of the male politician more than does the parody of the female, and that negative emotions and evaluations are higher in male condition, showing that politics, as today, is definitely more of a masculine domain (Lorenzi-Cioldi, 1988; Burr, 2002): therefore the aggressive act of parody affects the moral image of male politicians. On the contrary in the deep parody toward the female politician participants feel less indignation and evaluate her as less astute.

## Conclusion

Parody is a communicative act aimed at social criticism, performed by taking a person as Target, by finding out a trait or behavior denoting a relevant dèfaillance with respect to moral, esthetical, utilitarian criteria, and by making fun of it, thus pointing out and re-inforcing socially shared evaluation criteria. Different from serious criticism, by using the weapon of humor the parody induces positive emotions, amusement and laughter, and like any humorous performance triggers subtle cognitive processes necessary to catch the frame shift and the cognitive incongruity that evokes laughter. Yet, as our work demonstrates, while viewing a surface parody one is induced to a light level of frame shift, being exposed to a deep parody requires a more radical change of perspective that forces the Addressee to climb to a higher level of generality; this ends up with attributing the negative evaluation borne by the parody not only to its specific Target – for example, to that specific politician – but to a superordinated category that includes him/her but goes beyond. This might account for the more pervasive effect of negative emotions and evaluations we found in our data.

The deep parody, then, beside requiring more sophisticated processing in both production and comprehension, also has a deeper impact on the persuasive side, and thus the laughter it elicits has a bitter flavor.

A first limitation of our work is that it does not explicitly state the relation of types of parody and consequent emotions with behavioral intentions, for example potential voting intentions, and political participation. The investigation by Lee and Kwak (2014), who evidenced a relationship among political satire, experienced negative emotions, and intentions for political participation, will be replicated in future studies concerning the types of parody. A further limitation is the time frame of our experiment: we found out the changes in Character and Real Politician evaluation after seeing a video, but what is the duration of such effects? Can such a short-term manipulation produce long-term effects? Longitudinal studies are needed.

One more limitation is that our manipulation regarded an evaluation criterion – and corresponding discredit – focused on the dimension of benevolence: the Character’s reliability. But what differences might emerge should we consider discredit on esthetical, cognitive, or power criteria (D’Errico and Poggi, 2012)?

A final problem is that the political orientation of our participants was more frequently opposite to one of the parodied politician. However, the politician used as stimulus belonged to a somewhat distant period, thus no longer being representative of his electors, nor so highly beloved by them.

On the basis of the present studies, future research would need to draw a theoretical modeling of the effects of parody by considering different possible individual variables of the politician (right vs. left party, with respect to the gender and sexual orientation, present vs. past politician; representative or not), or of the target of parody criticism (dominance, competence, benevolence) and other contextual variables (historical period, and so on).

As proposed in previous works (Poggi et al., 2011; D’Errico and Poggi, 2013), a possible goal in studying the mechanisms of parody could be the construction of an “Artificial Parodist,” that is, an Embodied Agent, a little Robot, or a wearable system, that, through simulating the capabilities of a Human Parodist, might fulfill various functions: a Comedians’ Trainer, able to understand others’ goofy behaviors, discover their involuntary humor, and advice or teach how to make a parody of them; or a Parodist Teacher, able to make parodies of the pupils’ performance, for instance in choir singing (Poggi, forthcoming) or foreign language learning, to explain where they are wrong; or finally a wearable Parodist Companion, capable, when perceiving the User’s goofy behaviors, of making a parody of him, to teach him not to take himself too seriously, recommend him more adaptive behavior, and thus prevent him from being ridiculed by others.

While those works were mainly focused on the processes of parody production by a Parodist, the goal of the present paper was to shed light on the reception of this form of communication: more specifically, on the mechanisms through which a particular arrangement of information in a parody can elicit specific affective, evaluative, and persuasive processes in Addressees. On this side, a possible application of this study might rather be in the domain of persuasive technology (Fogg, 2002).

Research on socially aware systems requires fine-grained knowledge of the mechanisms of persuasion and social influence: in this perspective, investigation on parody, its processes of production and comprehension, and their effects on the Audience’s emotions and evaluations, can shed light on important aspects of social interaction and social cognition. For example, a tutoring system with civic purposes and thus with the goal of promoting deep political information in their audience/readers, can, by means of parody, amuse and at the same time affect their critical thoughts and political awareness in different ways depending on the types of representation and on the types of parody performed.

## Ethics Statement

Uninettuno University Ethics committee has approved the study on 27 January 2016. Uninettuno Ethics commission based the evaluation on Ethical Principles involving Human participants and on AIP ethics (Italian psychology association).

## Author Contributions

The two authors are responsible for the ideation of the whole article. FD is in particular responsible for collection, analysis and interpretation of data and results. IP is responsible for ideation and drafting the introduction and theoretical part. They both ensure the integrity and accuracy of the work.

## Conflict of Interest Statement

The authors declare that the research was conducted in the absence of any commercial or financial relationships that could be construed as a potential conflict of interest.
